# Attenuated Monocyte Apoptosis, a New Mechanism for Osteoporosis Suggested by a Transcriptome-Wide Expression Study of Monocytes

**DOI:** 10.1371/journal.pone.0116792

**Published:** 2015-02-06

**Authors:** Yao-Zhong Liu, Yu Zhou, Lei Zhang, Jian Li, Qing Tian, Ji-Gang Zhang, Hong-Wen Deng

**Affiliations:** 1 Department of Biostatistics and Bioinformatics, Tulane University School of Public Health and Tropical Medicine, New Orleans, United States of America; 2 Center of System Biomedical Sciences, University of Shanghai for Science and Technology, Shanghai, China; Nanjing Medical University, CHINA

## Abstract

**Background:**

Osteoporosis is caused by excessive bone resorption (by osteoclasts) over bone formation (by osteoblasts). Monocytes are important to osteoporosis by serving as progenitors of osteoclasts and produce cytokines for osteoclastogenesis.

**Aim:**

To identify osteoporosis-related genes, we performed microarray analyses of monocytes using Affymetrix 1.0 ST arrays in 42 (including 16 pre- and 26 postmenopausal) high hip BMD (bone mineral density) vs. 31 (including 15 pre- and 16 postmenopausal) low hip BMD Caucasian female subjects. Here, high vs. low BMD is defined as belonging to top vs. bottom 30% of BMD values in population.

**Method:**

Differential gene expression analysis in high vs. low BMD subjects was conducted in the total cohort as well as pre- and post-menopausal subjects. Focusing on the top differentially expressed genes identified in the total, the pre- and the postmenopausal subjects (with a p <5E-03), we performed replication of the findings in 3 independent datasets of microarray analyses of monocytes (total N = 125).

**Results:**

We identified (in the 73 subjects) and successfully replicated in all the 3 independent datasets 2 genes, DAXX and PLK3. Interestingly, both genes are apoptosis induction genes and both down-regulated in the low BMD subjects. Moreover, using the top 200 genes identified in the meta-analysis across all of the 4 microarray datasets, GO term enrichment analysis identified a number of terms related to induction of apoptosis, for which the majority of component genes are also down-regulated in the low BMD subjects. Overall, our result may suggest that there might be a decreased apoptosis activity of monocytes in the low BMD subjects.

**Conclusion:**

Our study for the first time suggested a decreased apoptosis rate (hence an increased survival) of monocytes, an important osteoclastogenic cell, as a novel mechanism for osteoporosis.

## Introduction

Osteoporosis is a prevalent bone metabolic disease characterized by bone fragility and an increased risk of low trauma fractures, especially in elderly females. As a key pathophysiological mechanism, the disease is caused by excessive bone resorption (by osteoclasts) over bone formation (by osteoblasts). Here, a low trauma fracture is defined as a fracture resulting from a fall from a standing position or lower [1], which is in contrast to a high trauma fracture defined as a fracture due to motor vehicle accidents or falls from greater than a standing height [2].

Genetics plays an important role in development of osteoporosis, as evidenced by a high heritability (~60%) of bone mineral density (BMD) [3–5], a key phenotype for osteoporosis. Over the last decade, a large number of genes have been identified for osteoporosis through a variety of genetics and genomics approaches [6].

Peripheral blood monocytes (PBMs) are a nice model for osteoporosis study. They are osteoclast progenitor cells [7–10] and produce cytokines for osteoclastogenesis and bone resorption [11,12]. Through PBMs gene/protein expression studies, a number of novel genes/proteins and new pathophysiological mechanisms have been identified for osteoporosis [13–17], which expanded our understanding of this complex disease. These studies demonstrated that genomic analyses of PBMs may reveal genes for osteoporosis at the osteoclast progenitor stage.

Here with a sample of 73 Caucasian females, stratified by hip BMD and menopause status, we performed a new microarray-based transcriptome-wide gene expression study of osteoporosis using the latest Affymetrix Human Exon 1.0 ST arrays. Our working hypothesis is that those genes differentially expressed in PBMs in high vs. low BMD subjects may underlie BMD variation. To replicate the findings from the 73 samples, we performed *in silico* replication using 3 existing monocyte microarray datasets for osteoporosis study [13,15,16]. We also performed meta-analysis across all the four microarray datasets and selected the top 200 genes with the most significant meta-analysis p values for GO (gene ontology) term enrichment analysis. In addition, we conducted verification of the identified genes using osteoporosis GWAS datasets, including the largest osteoporosis GWAS meta-analysis dataset, the GEFOS [18].

## Materials and Methods

### Subjects for the microarray study

The study was approved by Institutional Review Board or Research Administration of the involved institutions. Signed informed-consent documents were obtained from all study participants before entering the study. Exclusion criteria were used to exclude the comorbidities of osteoporosis from this study, e.g., severe diabetes (that can only be controlled with insulin).

Our discovery cohort (discovery dataset) contains 73 Caucasian females, with the age from 47 to 56. The sample is stratified by hip BMD and menopausal status. Their detailed characteristics are presented in Table 1. In brief, the high BMD group contains 42 subjects, with 16 pre- and 26 postmenopausal subjects. The low BMD group contains 31 subjects, with 15 pre- and 16 postmenopausal subjects. Menopause is defined as cessation of regular menses for at least one year. The raw microarray data for this cohort were submitted to GEO (Gene Expression Omnibus) under the accession number GSE56814.

**Table 1:** Basic characteristics of discovery and replication cohorts for monocyte microarray analyses.

Discovery cohort (73 Caucasian females)						
Menopausal status	High BMD			Low BMD		
	N	Age	Hip BMD Z score	N	Age	Hip BMD Z score
Premenopausal	16	51.0 (1.8)	1.54 (0.52)	15	50.0 (2.0)	-0.93 (0.36)
Postmenopausal	26	54.0 (1.8)	1.28 (0.46)	16	52.6 (2.5)	-1.17 (0.60)
Total	42	52.9 (2.3)	1.38 (0.49)	31	51.4 (2.6)	-1.05 (0.51)
Replication cohort I (80 Caucasian females)						
Menopausal status	High BMD			Low BMD		
	N	Age	Hip BMD Z score	N	Age	Hip BMD Z score
Premenopausal	20	41.7 (1.8)	1.32 (0.66)	20	42.3 (1.8)	-1.07 (0.51)
Postmenopausal	20	57.2 (1.9)	1.58 (0.66)	20	57.6 (1.5)	-1.04 (0.38)
Total	40	49.4 (8.1)	1.45 (0.67)	40	50.0 (7.9)	-1.05 (0.44)
Replication cohort II (19 Caucasian females)						
Menopausal status	High BMD			Low BMD		
	N	Age	Hip BMD Z score	N	Age	Hip BMD Z score
Premenopausal	5	49.4 (3.2)	2.71 (0.90)	4	50.5 (2.9)	-1.11 (0.43)
Postmenopausal	5	52.6 (1.8)	2.22 (0.93)	5	51.4 (1.5)	-1.69 (0.16)
Total	10	51.0 (3.0)	2.47 (0.90)	9	51.0 (2.1)	-1.43 (0.42)
Replication cohort III (26 Chinese females)						
Menopausal status	High BMD			Low BMD		
	N	Age	Hip BMD Z score	N	Age	Hip BMD Z score
Premenopausal	14	28.7 (4.7)	1.57 (0.57)	12	25.3 (3.1)	-1.72 (0.60)

Note: Age and hip BMD Z score are shown as mean (standard deviation).

For replication purpose, 3 independent cohorts were involved in this study, which were used in several previous monocyte microarray studies for osteoporosis [13,15,16]. Replication cohort I contains 80 Caucasian females, including 40 high (20 pre- and 20 postmenopausal) and 40 low hip BMD (20 pre- and 20 postmenopausal) subjects [16]. Replication cohort II contains 19 Caucasian females, including 10 high (5 pre- and 5 postmenopausal) and 9 low hip BMD (4 pre- and 5 postmenopausal) subjects [13]. Replication cohort III contains 26 Chinese females, all premenopausal and including 14 high and 12 low hip BMD subjects [15]. Definition of menopause for replication cohorts I and II is the same as for the discovery cohort. The basic characteristics for the 3 replication cohorts are also presented in Table 1. The raw microarray data for Replication cohorts I to III are archived in GEO under accession numbers GSE56815, GDS1287 and GSE7158, respectively.

### BMD measurements

For our discovery cohort, hip BMD (g/cm^2^) was measured using a Hologic dual energy x-ray absorptiometer (DXA) scanner (Hologic Corp., Waltham, MA). The machine was calibrated daily. The coefficient of variation of the dual energy x-ray absorptiometer measurements for BMD was 0.9%. None of our subjects had any known conditions that might artificially increase BMD values, such as osteophytes and facet sclerosis. The obtained BMD value was then transformed into a Z score, which is the units of standard deviations above or below the mean of a healthy, ethnic-, gender-, and age-matched reference population.

We used the same method for BMD measurement for the 3 replication cohorts. The detailed description can be found in the published studies using the three cohorts [13,15,16].

### Experimental procedures

The experimental procedures for our discovery cohort samples are briefly described as follows. The corresponding procedures for our replication cohort samples were similar and are described in the published studies using the samples [13,15,16].

**Monocyte isolation**. Monocyte isolation from whole blood was performed with a monocyte-negative isolation kit (Miltenyi Biotec Inc, Auburn, CA) following manufacturer's recommendation.

**Total RNA extraction**. Total RNA from monocytes was extracted using Qiagen RNeasy Mini kit (Qiagen, Inc., Valencia, CA). We used Agilent Bioanalyzer (Agilent, Santa Clara, CA) to control the RNA quality before each array experiment, where RNA integrity number (RIN) should be no less than 7.0 [19,20].

**Array experiments**. Preparation of cDNA, hybridization, and scanning of the GeneChip Human Exon 1.0 ST Array was performed according to the manufacturer's protocol for the Exon 1.0 Array (Affymetrix, Santa Clara, CA), which was published online via Affymetrix’s website: http://www.affymetrix.com/estore/catalog/131452/AFFY/Human+Exon+ST+Array#1_3.

### Microarray data analysis

On both the discovery cohort and replication cohort datasets, we used RMA (robust multiarray average) method [21] to normalize the array signals through the Bioconductor’s Oligo package [22]. After normalization of a certain dataset, we performed differential expression analysis using *t* test through the Bioconductor’s LIMMA (linear models for microarray data) package [23–25].

### GO term enrichment analysis using DAVID

We performed GO (gene ontology) term enrichment analysis using DAVID (Database for Annotation, Visualization and Integrated Discovery) v6.7 software package (http://david.abcc.ncifcrf.gov/) [26]. Our specific focus is on apoptosis-induction-related GO terms to see if these terms are significantly enriched.

To select those genes that are submitted to DAVID for enrichment analysis, we performed meta-analysis across all the four microarray datasets. We first selected those genes that have the same direction of regulation (i.e., down- or up-regulation in low BMD subjects) across all the four datasets. On those selected genes we then performed meta-analysis using Fisher’s method [27,28] to summarize the p values across all the four datasets. We then ranked the meta-analysis p values and selected the top 200 genes with the most significant p values. These 200 genes were then submitted to DAVID software for enrichment analysis.

The above analyses (gene selection, meta-analysis and enrichment analysis) were also performed in pre- and postmenopausal subjects separately across all the four microarray datasets.

### GWAS datasets confirmation

We took advantage of 7 GWAS datasets, either from our own group or selected from dbGAP (the Database of Genotype and Phenotype, http://www.ncbi.nlm.nih.gov/gap/, to confirm several genes (DAXX, PLK3, PDCD5 and VDAC1) for association with hip BMD at the population level. The related information is detailed as follows.

**GWAS datasets**. Seven GWAS datasets were involved in the analysis, of which 3 were from our own studies and 4 were selected from the dbGAP. All the studies related to the datasets were approved by the respective institutional ethics review boards and all participants provided written informed consent.

Our own GWAS datasets include: 1) OOS (Omaha osteoporosis study) [29] with 987 unrelated Caucasians; 2) KCOS (Kansas City Osteoporosis Study) with 2,250 unrelated Caucasians; and 3) COS (Chinese Osteoporosis Study) with 1,547 unrelated Chinese of Han ethnicity. The basic characteristics of the 3 cohorts are shown in Table 2.

**Table 2:** Basic characteristics of 7 GWAS cohorts.

Cohorts	N	Population	Female (%)	Age (yrs)	Height (m)	Weight (kg)	Hip BMD (g/cm^2^)	Bone densitometer used
OOS	987	Caucasian	49.6	50.3 (18.3)	1.71 (0.10)	80.10 (17.72)	0.97 (0.16)	Hologic QDR 4500W
KCOS	2250	Caucasian	75.9	51.4 (13.8)	1.66 (0.08)	75.16 (17.47)	0.97 (0.17)	Hologic QDR 4500W
COS	1547	Han Chinese	50.7	34.8 (13.4)	1.64 (0.08)	60.27 (10.54)	0.92 (0.13)	Hologic QDR 4500W
FHS	3747	Caucasian	57.3	60.3 (10.7)	1.66 (0.10)	77.00 (16.99)	0.95 (0.17)	Lunar DPX-L
IFS	1488	Caucasian	100.0	32.7 (7.2)	1.65 (0.06)	71.66 (16.90)	−	Lunar DPX-L
WHI-AA	712	African American	100.0	60.9 (6.9)	1.62 (0.06)	83.15 (17.72)	0.95 (0.15)	Hologic QDR-2000
WHI-HIS	409	Hispanic	100.0	60.7 (7.2)	1.57 (0.06)	73.87 (15.62)	0.86 (0.13)	Hologic QDR-2000

Note: 1. Age, height, weight and hip BMD data are presented as mean (standard deviation).

2. Raw hip BMD values are not available in the dbGAP for the IFS cohort, for which there are only adjusted hip BMD values in the dbGAP. In the analysis for this cohort, we therefore used adjusted hip BMD values directly.

The 4 dbGAP datasets include: 1) FHS (Framingham Heart Study), a longitudinal and prospective cohort comprising over 16,000 individuals of European ancestry spanning three generations [30]. Focusing on the first two generations, we identified 3,747 phenotyped individuals. 2) IFS (Indiana Fragility Study), a cross-sectional cohort comprising 1,493 premenopausal sister pairs of European ancestry [18]. After quality control, 1,488 subjects were selected for analysis. 3) WHI-AA (Women’s Health Initiative, African American ancestry): WHI is an observational study of a partial factorial randomized and longitudinal cohort with over 12,000 genotyped women aging 50–79 years, of African-American or Hispanic ancestry [31]. The WHI-AA dataset contains 712 phenotyped individuals of African-American ancestry. 4) WHI-HIS (WHI, Hispanic ancestry): containing 409 phenotyped individuals of Hispanic ancestry from the WHI cohort. The basic characteristics of the above 4 cohorts are shown in Table 2.

**Phenotype measurements and modeling**. Hip BMD was measured with DXA scanners following the manufacturer protocols; either Lunar scanners (Lunar Corp., Madison, WI, USA) or Hologic scanners (Hologic Inc., Bedford, MA, USA) were used for measuring individual cohorts. Covariates were screened among gender, age, age square, weight, and height, with the step-wise linear regression model. Raw hip BMD measurements were adjusted by significant covariates. To adjust for potential population stratification, the first five principal components derived from genome-wide genotype data were also included as covariates [32]. Residual phenotypes after adjustment were normalized by inverse quantile of the standard normal distribution to impose a normal distribution on phenotypes that were analyzed subsequently [33].

**Genotyping and quality control (QC)**. All the GWAS cohorts were genotyped by high-throughput SNP genotyping arrays (Affymetrix Inc., Santa Clara, CA; or Illumina Inc., San Diego, CA, USA for individual cohorts) following the manufacturer’s protocols. QC of genotype data were implemented with Plink [34], with the following criteria applied: individual missingness < 5%, SNP call rate > 95%, and Hardy-Weinberg equilibrium (HWE) p-value > E-05. For familial samples (FHS and IFS), all genotypes with Mendel error were set to missing.

**Association testing**. Each GWAS cohort was tested for association between hip BMD and SNPs under an additive mode of inheritance. For unrelated samples, association was examined by the linear regression model with MACH2QTL [35], in which allele dosage was taken as the predictor for the phenotype. For familial samples (FHS and IFS), a mixed linear model was used in which the effect of genetic relatedness within each pedigree was also taken into account [36]. Genomic control inflation factor was estimated for each individual GWAS [37].

**Meta-analysis**. Summary statistics of associations from each GWAS were combined to perform weighted fixed-effect meta-analysis with METAL [38], in which weights were proportional to the square-root of each sample size. Cochran’s Q statistic and I^2^ were calculated as measures of between-study heterogeneity [39].

### GEFOS replication

We checked the SNPs of the four apoptosis genes (DAXX, PLK3, PDCD5 and VDAC1) for their association signals in the GEFOS (Genetic factors for osteoporosis consortium) GWAS datasets, which were downloaded from www.gefos.org. The datasets include GWAS meta-analysis p values for >2 million SNPs for association with femoral neck (in male, female and all subjects) and lumbar spine BMD (for male, female and all subjects).

### Real-time PCR confirmation

We performed real-time RT-PCR in a subset of the discovery cohort samples (n = 51). The samples are made up of 30 high BMD subjects and 21 low BMD subjects, among whom, 13 are premenopausal high BMD subjects, 17 postmenopausal high BMD subjects, 10 premenopausal low BMD subjects and 11 postmenopausal low BMD subjects. To confirm differential expression for the VDAC1 gene, the experiment was performed in the total 51 subjects since the differential expression for this gene was discovered by microarray in the total discovery cohort. To confirm differential expression for the DAXX, PLK3 and PDCD5 genes, the experiment was performed in the 13 high vs. 10 low BMD premenopausal subjects since the differential expression for the three genes was discovered by microarray in the premenopausal subjects of the discovery cohort. For the experiment, we used TaqMan Assays and StepOne Plus Real-Time PCR system from Life Technologies (Grand Island, NY). We used GAPDH as the house-keeping gene. The experimental data were analyzed using the StepOne Software v.2.2.2, which produced a ∆CT value as a quantitative measure for the target gene transcript abundance for each sample. We used the ∆CT value from each sample and performed student *t* test to compare the expression levels in samples from high vs. low BMD subjects.

### Other analyses

Using the NIH SNP Function Prediction database (http://snpinfo.niehs.nih.gov/snpinfo/ snpfunc.htm), we checked the potential functions for the SNPs nearby the two genes, which were replicated in the 7 GWAS datasets for association with hip BMD or associated with BMD in the GEFOS dataset.

Using SPSS (IBM, Armonk, NY), we performed normality test for the ∆CT values from the real-time PCR experiment used for the t test.

A classical pathway for osteoblast precipitated osteoclast activity is through RANKL-RANK and RANKL-OPG interactions [40]. Using UniHI7, a database for human molecular interaction networks [41], we retrieved the potential interacting partners of RANKL, RANK and OPG.

## Results

In the discovery cohort (n = 73) microarray dataset (GSE56814), we identified a large number of genes differentially expressed (with p < 0.05) in high (n = 42) vs. low BMD (n = 31) subjects, in premenopausal high (n = 16) vs. low BMD (n = 15) subjects, and in postmenopausal high (n = 26) vs. low (n = 16) BMD subjects. To avoid false positive findings, we focused only on the top well-annotated differentially expressed genes (which have a p value of < 5E-03) identified in the total group as well as in the subgroup (pre- or postmenopausal groups) analyses and performed replication analysis to validate these genes’ differential expression in terms of BMD in our replication cohort datasets. By successful replication, we mean that a gene must achieve a p value </ = 0.10 in a replication cohort dataset with the same direction of effect in both the discovery and replication cohort datasets (e.g., upregulated in low BMD subjects in both the discovery and replication cohorts).

Using replication cohorts I (n = 80) (GSE56815), II (n = 19) (GDS1287) and III (n = 26) (GSE7158), we successfully replicated 2 genes (DAXX and PLK3). They are both apoptosis induction genes [42,43] and both down-regulated in the low BMD subjects. The p values and direction of regulation for the 2 genes in our discovery and replication cohorts are listed in Table 3. We also used Fisher’s method [27,28] for meta-analysis to combine the p values achieved in the discovery and replication cohort datasets and the meta-analysis p values are also shown in the table.

**Table 3:** Apoptosis genes identified and replicated in microarray analysis of monocytes in high vs. low BMD subjects.

Gene symbol^1^	P value				Meta-analysis p value^2^	Comparison groups^3^ in discovery cohort and replication cohort I	Up- or down-regulated in low BMD group
	Discovery cohort	Replication cohort I	Replication cohort II	Replication cohort III			
DAXX	1.44E-04	8.20E-03	5.72E-02	6.96E-02	6.48E-06	premenopausal	down
PLK3	4.75E-03	5.55E-02	1.16E-02	8.97E-02	1.94E-04	premenopausal	down
PDCD5	6.39E-05	1.42E-02	NS^4^	NS	1.35E-05	premenopausal	down
VDAC1	8.32E-05	2.61E-02	NS	NS	3.05E-05	total group	down

Note: 1. Full names of the genes: DAXX (DEATH-ASSOCIATED PROTEIN 6), PLK3 (POLO-LIKE KINASE 3), PDCD5 (PROGRAMMED CELL DEATH 5), VDAC1 (VOLTAGE-DEPENDENT ANION CHANNEL 1)

2. The meta-analysis p values summarize the p values in the discovery and the replication cohorts using Fisher’s method [27,28].

3. Comparison group means that the differential expression analysis was performed in the total or a subgroup (e.g., premenopausal group). For replication cohorts II and III, the differential expression analysis was performed in the total group.

4. NS: non-significant.

In Table 3, we also include 2 other apoptosis induction genes, PDCD5 [44,45] and VDAC1 [46,47], which are replicated in the largest replication cohort, replication cohort I (n = 80). In total, there are 98 genes replicated in replication cohort I, and PDCD5 and VDAC1 are ranked at 7^th^ and 17^th^, respectively, according to their meta-analysis p values. The detailed information of these 98 replicated genes is included in S1 Table.

We selected those genes that have the same direction of regulation (i.e., down- or up-regulation in low BMD subjects) across all the four microarray datasets and performed meta-analysis across the four datasets on the selected genes using Fisher’s method [27,28]. Those top 200 genes with the most significant meta-analysis p values were submitted to DAVID (Database for Annotation, Visualization and Integrated Discovery) v6.7 software package (http://david.abcc.ncifcrf.gov/) [26] for GO term enrichment analysis. The above analyses (gene selection, meta-analysis and enrichment analysis) were performed in both the total subjects as well as pre- and postmenopausal subjects separately across all the four microarray datasets.

Through the analysis, we identified five significantly enriched GO terms (enrichment p values ranging from 6.90E-04 to 6.40E-03) that are related to induction of apoptosis in the premenopausal subjects, i.e., GO:0006917~induction of apoptosis (p = 6.90E-04), GO:0012502~induction of programmed cell death (p = 7.09E-04), GO:0043065~positive regulation of apoptosis (p = 1.01E-03), GO:0043068~positive regulation of programmed cell death (p = 1.08E-03) and GO:0010942~positive regulation of cell death (p = 1.12E-03). More detailed information for these GO terms is presented in Table 4. Interestingly, among the 15 component genes for these GO terms, the majority (9 genes) were down-regulated in the low BMD subjects, suggesting an overall trend of down-regulation of apoptosis-induction in the low BMD subjects, which is consistent with the pattern as suggested in the four individual apoptosis genes as discussed above (DAXX, PLK3, PDCD5 and VDAC1).

**Table 4:** GO term enrichment analysis using DAVID.

GO Term	Enrichment P value	Component genes in GO term
GO:0006917~induction of apoptosis	6.90E-04	**PPP2R1A**, BCLAF1, **ABR**, **TP63**, **ITSN1**, PTEN, **DAXX**, **TGFB1**, **HMOX1**, SOS2, MAPK9, JAK2, CASP1
GO:0012502~induction of programmed cell death	7.09E-04	**PPP2R1A**, BCLAF1, **ABR**, **TP63**, **ITSN1**, PTEN, **DAXX**, **TGFB1**, **HMOX1**, SOS2, MAPK9, JAK2, CASP1
GO:0043065~positive regulation of apoptosis	1.01E-03	**PPP2R1A**, BCLAF1, **ABR**, **TP63**, **ITSN1**, PTEN, **DAXX, TGFB1**, **AKT1**, **HMOX1**, SOS2, **PPP3CC**, MAPK9, JAK2, CASP1
GO:0043068~positive regulation of programmed cell death	1.08E-03	**PPP2R1A**, BCLAF1, **ABR**, **TP63**, **ITSN1**, PTEN, **DAXX, TGFB1, AKT1, HMOX1**, SOS2, **PPP3CC**, MAPK9, JAK2, CASP1
GO:0010942~positive regulation of cell death	1.12E-03	**PPP2R1A**, BCLAF1, **ABR**, **TP63, ITSN1**, PTEN, **DAXX**, **TGFB1**, **AKT1, HMOX1**, SOS2, **PPP3CC**, MAPK9, JAK2, CASP1

Note: 1. The following genes (bolded in the table) are down-regulated in the low BMD subjects with meta-analysis p values ranging from 3.50E-06 to 3.24E-04: ITSN1, TP63, DAXX, PPP2R1A, PPP3CC, ABR, HMOX1, AKT1, TGFB1

2. The following genes in the table are up-regulated in the low BMD subjects with meta-analysis p values ranging from 3.59E-06 to 3.32E-04: BCLAF1, JAK2, SOS2, CASP1, MAPK9, PTEN

We also submitted to DAVID for enrichment analysis the 98 differentially expressed genes that were identified in the discovery cohort and replicated in the replication cohort I (S1 Table). Through DAVID analysis of these genes, we identified a GO term “cell cycle arrest (GO:0007050)”, which achieved a p value of 2.60E-04. The term contains six genes (HBP1, CUL1, CDKN2D, GAS2L1, PKD1, PPP1R15A), among which, five genes (HBP1, CDKN2D, GAS2L1, PKD1, PPP1R15A) are down-regulated in low vs. high BMD subjects (S1 Table), suggesting an overall decreased cell cycle arrest (i.e., increased proliferation) in low vs. high BMD subjects. Therefore, our data also supported “increased monocyte hyperplasia” in low BMD vs. high BMD subjects.

Using the 7 GWAS datasets, we assessed for association with hip BMD at the population level for the 4 apoptosis genes, among which, 3 genes, VDAC1, DAXX and PLK3, appear to be associated with hip BMD. The 3 genes have SNPs achieving p values of < 0.05 according to meta-analysis over the 7 GWAS datasets. The detailed information of the SNPs is presented in Table 5. No between-study heterogeneity was detected for the SNPs as shown in the table by their low I^2^ (<50%) and high Q p-values (>0.05).

**Table 5:** Meta-analysis results for 3 apoptosis genes for association with hip BMD in the 7 GWAS datasets.

Gene	SNP name	SNP location	Allele^1^	Minor allele frequency^2^	Meta-analysis p value	I^2^	Q p-value
VDAC1	rs34133155	Intron	G/T	0.103/0.016/0.138	2.88E-03	12%	0.34
DAXX	rs11873	Exon	A/G	0.013/0.007/0.016	2.41E-02	0	0.91
PLK3	rs17883049	Intron	A/G	0.176/0.150/0.253	4.26E-02	0	0.53

Note: 1. The first allele shown in the table is the minor allele.

2. For minor allele frequency (MAF), the three frequencies provided are the average, smallest, and largest MAFs detected in the 7 GWAS study populations.

In the GEFOS dataset, the SNPs from the DAXX, PLK3 and VDAC1 genes also achieved signals of association with BMD phenotypes. The results are summarized in Table 6. Four SNPs of DAXX, rs1059231 (synonymous variant), rs2073524 (intron variant), rs2073525 (upstream gene variant) and rs2239839 (intron variant), achieved p values of 3.26E-02, 4.52E-03, 8.19E-03 and 3.02E-02, respectively, for association with femoral neck BMD in men. Three SNPs of PLK3, rs6676749 (upstream gene variant), rs11211036 (synonymous variant) and rs11584440 (intron variant), achieved p values of 1.14E-02, 1.40E-02 and 1.16E-02, respectively, for association with femoral neck BMD in men.

**Table 6:** Results for 3 apoptosis genes for association with BMD in the GEFOS dataset.

SNP name	SNP location	Gene	Association p value	Associated bone phenotype	Gender group
rs1059231	exon	DAXX	3.26E-02	femoral neck BMD	men
rs2073524	intron	DAXX	4.52E-03	femoral neck BMD	men
rs2073525	upstream	DAXX	8.19E-03	femoral neck BMD	men
rs2239839	intron	DAXX	3.02E-02	femoral neck BMD	men
rs6676749	upstream	PLK3	1.14E-02	femoral neck BMD	men
rs11211036	exon	PLK3	1.40E-02	femoral neck BMD	men
rs11584440	intron	PLK3	1.16E-02	femoral neck BMD	men
rs6880980	intron	VDAC1	2.40E-02	femoral neck BMD	women
rs6880980	intron	VDAC1	7.69E-03	lumbar spine BMD	women
rs6893145	intron	VDAC1	2.68E-02	femoral neck BMD	women
rs6893145	intron	VDAC1	7.80E-02	lumbar spine BMD	women
rs6897409	intron	VDAC1	2.40E-02	femoral neck BMD	women
rs6897409	intron	VDAC1	7.94E-02	lumbar spine BMD	women
rs7404	3’ UTR	VDAC1	9.71E-03	lumbar spine BMD	women
rs2288834	upstream	VDAC1	5.35E-04	lumbar spine BMD	women
rs4279383	downstream	VDAC1	3.18E-02	lumbar spine BMD	women
rs6878448	intron	VDAC1	7.18E-04	lumbar spine BMD	women
rs6878988	intron	VDAC1	5.16E-04	lumbar spine BMD	women

Eight SNPs of VDAC1 achieved association signals in the GEFOS dataset. In particular, rs6880980 (intron variant) achieved p values of 2.40E-02 and 7.69E-03, for association with femoral neck BMD in women and lumbar spine BMD in women, respectively. The SNP, rs6893145 (intron variant), achieved p values of 2.68E-02 and 7.80E-02 for association with femoral neck BMD in women and lumbar spine BMD in women, respectively. The SNP, rs6897409 (intron variant), achieved p values of 2.40E-02 and 7.94E-02 for association with femoral neck BMD in women and lumbar spine BMD in women, respectively. Five additional SNPs of VDAC1 also achieved association signals for lumbar spine BMD in women, which are rs7404 (3’ UTR variant) with a p value of 9.71E-03, rs2288834 (upstream gene variant) with a p value of 5.35E-04, rs4279383 (downstream gene variant) with a p value of 3.18E-02, rs6878448 (intron variant) with a p value of 7.18E-04 and rs6878988 (intron variant) with a p value of 5.16E-04.

Using the NIH SNP Function Prediction database (http://snpinfo.niehs.nih.gov/snpinfo/ snpfunc.htm), we checked the potential functions for the SNPs nearby the two genes DAXX and PLK3, including rs11873, rs1059231, rs2073524, rs2073525, and rs2239839 for DAXX, and rs17883049, rs6676749, rs11211036, and rs11584440 for PLK3, which were replicated in the 7 GWAS datasets for association with hip BMD (Table 5) or associated with BMD in the GEFOS dataset (Table 6).

Most SNPs achieved a Regulatory Potential Score [48] of >~0.30, which are 0.28 (for rs11873), 0.39 (for rs1059231), 0.31for (rs2073524), 0.45 (for rs2073525) for DAXX SNPs, 0.28 (for rs17883049), 0.31 (for rs11211036), and 0.41 (for rs11584440) for PLK3 SNPs. Some DAXX SNPs (rs2073524, rs2073525, rs2239839) and PLK3 SNPs (rs17883049, rs6676749) also contain transcriptional factor binding sites. Taken together, the results may suggest that these SNPs might have potential roles in regulating expression of the DAXX and PLK3 genes.

A classical pathway for osteoblast precipitated osteoclast activity is through RANKL-RANK and RANKL-OPG interactions [40]. Using UniHI7 [41], a database for human molecular interaction networks, we retrieved the potential interacting partners of RANKL, RANK and OPG. Among these retrieved interactions, two interacting partners of RANKL, i.e., CALCR and NFKBIA, were found differentially expressed in our discovery cohort and their differential expression was also replicated in the replication cohort I (S1 Table). The first gene, CALCR, is upregulated in low vs. BMD subjects and the 2^nd^ gene, NFKBIA, is down-regulated in low vs. high BMD subjects (S1 Table). These two genes’ potential interaction with RANKL was supported by previous studies [49,50] as well as additional bioinformatics analyses at gene co-expression and GO co-annotation levels [41].

**Fig 1:**
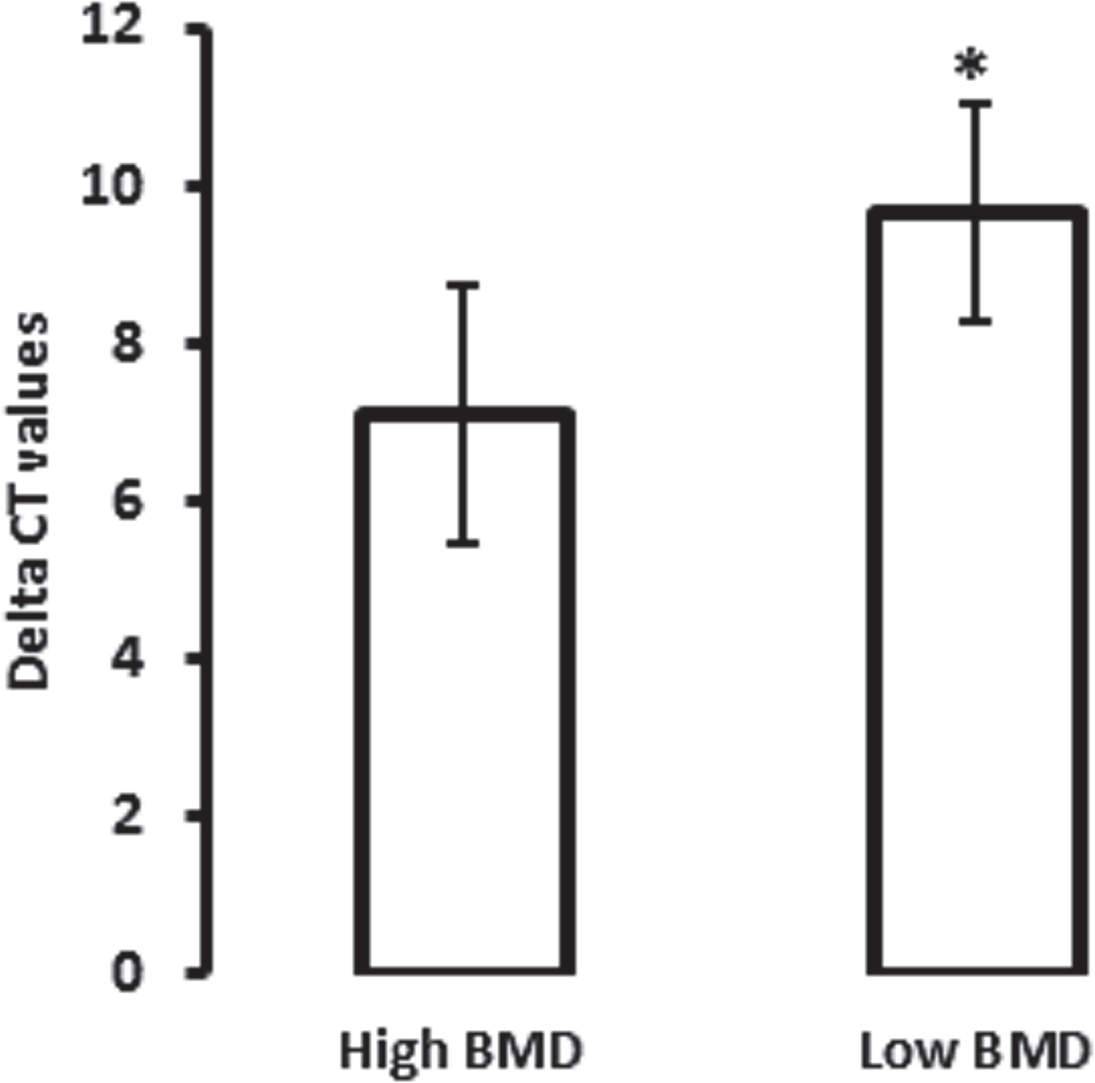
Real-time PCR result for the PLK3 gene. 1. *: Significant at the 0.05 level. 2. Error bars are for standard deviations. 3. The higher mean delta CT value in low vs. high BMD subjects indicates a lower average expression level in low vs. high BMD subjects.

Through real-time PCR and using a subset of the samples from our discovery cohort, we successfully confirmed the differential expression of the PLK3 gene in our premenopausal subjects’ samples (p = 0.016) based on the *t* test. The result is plotted in Fig. 1. As shown in Fig. 1, the higher mean delta CT value in low vs. high BMD subjects indicates a lower average expression level of the PLK3 gene in low vs. high BMD subjects.

Using SPSS (IBM, Armonk, NY), we performed normality test for the ∆CT values from the real-time PCR experiment used for the *t* test. For example, for the PLK3 gene, for which we performed *t* test and found statistical significance, the normality test based on the Shapiro-Wilk test did not reach significance (*p* = 0.280), suggesting that the ∆CT data do not deviate from normal distribution. Therefore, the *t* test should be appropriate for analysis of our real-time PCR data (the ∆CT values) since the data appear to follow a normal distribution.

We have not confirmed with real-time PCR the differential expression of DAXX, PDCD5 and VDAC1. It might be due to the fact that only a subset of the samples was used for real-time PCR confirmation, which decreased the power for detecting differential expression as in our microarray experiment. The reason that we did not use all the samples as those used in our microarray experiment is that some samples did not have sufficient total RNA for real-time PCR reactions. However, the differential expression of DAXX, PDCD5 and VDAC1 was supported across-cohort by other independent microarray datasets as shown in Table 3. Indeed, across-cohort replication using independent datasets may represent more convincing evidence than real-time PCR confirmation, which was performed only within-cohort.

## Discussion

We here report a transcriptome-wide gene expression study of monocytes for osteoporosis in 73 Caucasian females (the discovery cohort) with extremely high or low hip BMD. Focusing on the top differentially expressed genes (p < 5E-03) in high vs. low BMD subjects in the total, pre- and postmenopausal groups, we attempted to replicate the genes in 3 existing independent monocyte microarray datasets (the replication cohorts I, II and III) for osteoporosis study [13,15,16]. Two genes, DAXX and PLK3, were successfully replicated across all the 3 replication cohorts, with significant (p <0.05) or marginally significant (p <0.10) p values as well as the same direction of regulation as in our discovery cohort (Table 3). Impressively, the replication was achieved not only across cohort (in replication cohorts I and II, which are made up of Caucasians), but also across “ethnicity” (in replication cohort III that is made up of Chinese subjects). The result is significant since among ~20,000 human genes genome-wide under study only 2 stood out based on our stringent criteria for discovery and replication.

Importantly, the two replicated genes, DAXX and PLK3, are both apoptosis induction genes [42,43] and both down-regulated in the low BMD subjects. DAXX is a well-known gene for apoptosis. By binding to Fas death domain, it can enhance Fas-mediated apoptosis [42]. DAXX is also key to the apoptosis cascade initiated by other factors and pathways, such as TGF-beta [51] and ZIP kinase [52]. PLK3 is a member of the “polo” family of serine/threonine kinases [53]. Overexpression of PLK3 can lead to incomplete cytokinesis and apoptosis [54], which might be due to PLK3’s perturbation of microtubule integrity [43] and its function of centrosome localization [55] during cytokinesis.

This pattern of down-regulation of apoptosis induction genes in the low BMD subjects is also shown in those genes that were replicated in the largest replication cohort containing 80 subjects (S1 Table). Among the top 20 replicated genes (as ranked by meta-analysis p values), the 7^th^ and 17^th^ genes (PDCD5 and VDAC1, respectively) were also apoptosis genes, which were also down-regulated in the low BMD subjects. PDCD5 was found to be an early marker for apoptosis as its expression level was observed to be significantly increased in apoptotic cells and its nuclear translocation preceded cellular morphological changes of apoptosis, such as chromosome fragmentation [45]. VDAC1 plays an essential role in apoptosis in mammalian cells, in particular, the apoptosis signaling initiated by Bax-induced cytochrome c release from mitochondria [46,47]. When microinjected into cells, anti-VDAC antibodies were found to inhibit apoptosis of various forms [47].

Interestingly, through GO term enrichment analysis of the top 200 genes selected through meta-analysis of the 4 microarray datasets, we identified 5 GO terms related to induction of apoptosis (Table 4). The pattern of down-regulation of apoptosis induction in the low BMD subjects was again supported by the fact that among the 15 component genes for these 5 GO terms the majority (9 genes) were down-regulated in the low BMD subjects. In addition, through GO term enrichment analysis of the 98 genes identified in the discovery cohort and replicated the replication cohort I, we also identified a GO term “cell cycle arrest” (p = 2.60E-04) with the majority of the component genes (5 out of 6) down-regulated in low vs. high BMD subjects. Therefore, our data may also suggest “decreased cell cycle arrest” and hence “increased monocyte hyperplasia” in low BMD vs. high BMD subjects, which provided additional support to our primary findings of decreased monocyte apoptosis in low vs. high BMD subjects. Taken together, our data suggest a decreased monocyte apoptosis level as a novel mechanism for female osteoporosis.

Apoptosis of bone-related cells, including osteoclasts, osteoblasts and osteocytes, has been recognized as a key working mechanism for bone mass regulation and osteoporosis. For example, the well-known drug for osteoporosis, bisphosphonates, can inhibit pathologic bone resorption by inducing osteoclast apoptosis [56]. Another important osteoporosis-related agent, glucocorticoids, may cause bone loss by inhibiting osteoclast apoptosis [57] and stimulating osteoblast and osteocyte apoptosis [58]. Estrogen’s beneficial influence on bone may also be due to its pro-apoptotic effect on osteoclasts [59] and anti-apoptotic effect on osteoblasts [60]. To the best of our knowledge, our study is the first one to provide strong evidence for “reduced monocyte apoptosis” as another potential mechanism for osteoporosis. Given the known functions of monocytes in osteoclastogenesis [7–10] and inflammatory bone resorption [11,12], a decreased level of monocyte apoptosis (and a corresponding higher level of the cell’s survival), as suggested by our gene expression data in the low BMD subjects, may contribute to more active osteoclastogenesis and bone resorption, and therefore increase osteoporosis risk. Although the mechanism suggested by our study needs further validation, its soundness is supported by previous apoptosis studies on other types of bone cells and the well-established functions of monocytes.

Among the 4 microarray datasets we used in this study, the “most significant” genes are all different. This is not unexpected since in a genomic study, where a large number of genes are tested in parallel, the strongest signal (effect) is detected quite often due to overestimation of the effect size based on the sample itself rather than the population, a common phenomenon often referred as “winner’s curse” [61]. Because of that, the strongest hits in genomic studies are very difficult to replicate as those hits are quite often false positives. Due to the above consideration, our replication effort in this study does not focused on those strongest signals.

The four apoptosis genes as identified in our microarray studies are not among the list of osteoporosis genes as summarized in a Nature Reviews article [62] that reviewed the major GWAS of osteoporosis. Apart from the difficulty to replicate across studies due to an insufficient power to identify all of the causal genes at the same time in a study [63], the non-replication may also be due to the different approach used in this study (i.e., analysis at the gene expression level) from GWAS (i.e., analysis at the gene polymorphism level). Nevertheless, using the 7 GWAS datasets (Table 5) and the GEFOS dataset, we did confirm *in silico* association signals for the DAXX, PLK3 and VDAC1 genes, suggesting that the genes identified in this study may also contribute to BMD variation at the broad population level. In particular, the GEFOS is the largest dataset for osteoporosis GWAS meta-analysis [18]. It contains 57 distinct cohorts (http://www.gefos.org/?q=studies) for osteoporosis GWAS from Europe, Australia and Asia, with a total sample size of ~33,000 [18]. Therefore, the GEFOS data used in our *in silico* replication here are robust and may even represent a benchmark data for SNP association signals for osteoporosis.

We used BH (Benjamini Hochberg) method [64] to control FDR (false discovery rate) of the differentially expressed genes identified in the discovery cohort. Specifically, the four DEx genes (DAXX, PLK3, PDCD5 and VDAC1) replicated in several replication cohorts (Table 3) all achieved FDR values > 0.10 in our discovery cohort, which are not significant in terms of FDR. However, our major aim here is not to look at a transcript’s statistical significance in an individual dataset, but to take advantage of multiple datasets and check a transcript’s consistent signals across all the datasets. In terms of the latter aspect, these four genes are indeed significant as all of them achieved nominally significant (p < 0.05) or marginally significant (p < 0.10) p values in our discovery and replication datasets. In our study, we did not use FDR for selecting genes for replication since we would like to increase the likelihood (i.e., the power) for identifying genes that achieve consistent signals in multiple datasets, as the four genes shown in Table 3.

A classical pathway for osteoblast precipitated osteoclast activity is through RANKL-RANK and RANKL-OPG interactions [40]. Using UniHI7 [41], a database for human molecular interaction networks, we retrieved the potential interacting partners of RANKL, RANK and OPG. Interestingly, among these retrieved interactions, two interacting partners of RANKL, i.e., CALCR and NFKBIA, were found differentially expressed in our discovery cohort and their differential expression was also replicated in the replication cohort I (S1 Table). However, due to the descriptive nature of our data and the limited previous research in this field, the overall effects of these two genes’ (CALCR and NFKBIA) interaction with RANKL to osteoblast precipitated osteoclastogenesis cannot be fully determined in this study.

In summary, we have performed a systemic investigation of monocytes for osteoporosis risk. Our study, involving 4 independent microarray datasets for monocyte transcriptome-wide gene expression analyses, provides convincing evidence for the four apoptosis induction genes’ (DAXX, PLK3, VDAC1 and PDCD5) importance to BMD variation. So far, in the field of monocyte genomic analyses for osteoporosis, our study has the largest sample size and is the first one to perform replication across independent microarray datasets and also across ethnicity to support the discovery in our primary dataset, and hence the findings are robust. Moreover, the apoptosis genes identified were also confirmed by the GWAS signals from GEFOS [18], which further verified the genes’ relevance to osteoporosis risk. Above all, our findings suggested a novel mechanism for osteoporosis, which is attenuation of monocyte apoptosis, as supported by the 4 apoptosis genes’ (DAXX, PLK3, VDAC1 and PDCD5) down-regulation in the low BMD subjects. Our study highlights the functional importance of monocyte in bone metabolism and contributes to the novel insights into the genetic basis and pathophysiological mechanism of osteoporosis.

## Supporting Information

S1 Table Genes identified in the discovery cohort (n = 73) and replicated in the replication cohort I (n = 80). 1. Direction of regulation is the up- or down-regulation of a gene in the low BMD subjects.
2. Comparison group indicates the total, pre- or postmenopausal subgroups, where the differential expression is detected and replicated.
3. Meta-analysis p value was calculated by Fisher’s method [27,28] to combine the p values achieved in discovery cohort and replication cohort I. The genes in the table, 102 in total, are ranked by the meta-analysis p value.
4. PDCD5 is ranked at 7^th^.
5. VDAC1 is ranked at 17^th^
(DOCX)

## References

[1] Hider SL, Ismail A, Todd B, Rodgers J, Hamilton A, et al (2005) Does a patient-focused approach increase prescribing following a low trauma fracture? Rheumatology (Oxford) 44: 138–139. 15611316 10.1093/rheumatology/keh430

[2] Mackey DC, Lui LY, Cawthon PM, Bauer DC, Nevitt MC, et al (2007) High-trauma fractures and low bone mineral density in older women and men. JAMA 298: 2381–2388. 18042915 10.1001/jama.298.20.2381

[3] Dequeker J, Nijs J, Verstraeten A, Geusens P, Gevers G (1987) Genetic determinants of bone mineral content at the spine and radius: a twin study. Bone 8: 207–209. 3446256 10.1016/8756-3282(87)90166-9

[4] Pocock NA, Eisman JA, Hopper JL, Yeates MG, Sambrook PN, et al (1987) Genetic determinants of bone mass in adults. A twin study. J Clin Invest 80: 706–710. 3624485 10.1172/JCI113125 PMC442294

[5] Smith DM, Nance WE, Kang KW, Christian JC, Johnston CC Jr. (1973) Genetic factors in determining bone mass. J Clin Invest 52: 2800–2808. 4795916 10.1172/JCI107476 PMC302548

[6] Xu XH, Dong SS, Guo Y, Yang TL, Lei SF, et al (2010) Molecular genetic studies of gene identification for osteoporosis: the 2009 update. Endocr Rev 31: 447–505. 10.1210/er.2009-0032 20357209 PMC3365849

[7] Fujikawa Y, Quinn JM, Sabokbar A, McGee JO, Athanasou NA (1996) The human osteoclast precursor circulates in the monocyte fraction. Endocrinology 137: 4058–4060. 8756585 10.1210/endo.137.9.8756585

[8] Higuchi S, Tabata N, Tajima M, Ito M, Tsurudome M, et al (1998) Induction of human osteoclast-like cells by treatment of blood monocytes with anti-fusion regulatory protein-1/CD98 monoclonal antibodies. J Bone Miner Res 13: 44–49. 9443789 10.1359/jbmr.1998.13.1.44

[9] Matayoshi A, Brown C, DiPersio JF, Haug J, Abu-Amer Y, et al (1996) Human blood-mobilized hematopoietic precursors differentiate into osteoclasts in the absence of stromal cells. Proc Natl Acad Sci U S A 93: 10785–10790. 8855258 10.1073/pnas.93.20.10785 PMC38233

[10] Purton LE, Lee MY, Torok-Storb B (1996) Normal human peripheral blood mononuclear cells mobilized with granulocyte colony-stimulating factor have increased osteoclastogenic potential compared to nonmobilized blood. Blood 87: 1802–1808. 8634426

[11] Bell NH (2003) RANK ligand and the regulation of skeletal remodeling. J Clin Invest 111: 1120–1122. 12697730 10.1172/JCI18358 PMC152945

[12] Pacifici R (1996) Estrogen, cytokines, and pathogenesis of postmenopausal osteoporosis. J Bone Miner Res 11: 1043–1051. 8854239 10.1002/jbmr.5650110802

[13] Liu YZ, Dvornyk V, Lu Y, Shen H, Lappe JM, et al (2005) A novel pathophysiological mechanism for osteoporosis suggested by an in vivo gene expression study of circulating monocytes. J Biol Chem 280: 29011–29016. 15965235 10.1074/jbc.M501164200

[14] Deng FY, Liu YZ, Li LM, Jiang C, Wu S, et al (2008) Proteomic analysis of circulating monocytes in Chinese premenopausal females with extremely discordant bone mineral density. Proteomics 8: 4259–4272. 10.1002/pmic.200700480 18924182 PMC2760933

[15] Lei SF, Wu S, Li LM, Deng FY, Xiao SM, et al (2009) An in vivo genome wide gene expression study of circulating monocytes suggested GBP1, STAT1 and CXCL10 as novel risk genes for the differentiation of peak bone mass. Bone 44: 1010–1014. 10.1016/j.bone.2008.05.016 19223260

[16] Chen XD, Xiao P, Lei SF, Liu YZ, Guo YF, et al. (2009) Gene Expression Profiling in Monocytes and SNP Association Suggest the Importance of STAT1 Gene for Osteoporosis in Both Chinese and Caucasians. J Bone Miner Res. 10.1359/jbmr.090724 PMC3153389 19594299

[17] Deng FY, Lei SF, Zhang Y, Zhang YL, Zheng YP, et al. (2011) Peripheral blood monocyte-expressed ANXA2 geneis involved in pathogenesis of osteoporosis in humans. Mol Cell Proteomics. 10.1074/mcp.M111.011700 PMC3226411 21817168

[18] Estrada K, Styrkarsdottir U, Evangelou E, Hsu YH, Duncan EL, et al (2012) Genome-wide meta-analysis identifies 56 bone mineral density loci and reveals 14 loci associated with risk of fracture. Nat Genet 44: 491–501. 10.1038/ng.2249 22504420 PMC3338864

[19] Schroeder A, Mueller O, Stocker S, Salowsky R, Leiber M, et al (2006) The RIN: an RNA integrity number for assigning integrity values to RNA measurements. BMC Mol Biol 7: 3 16448564 10.1186/1471-2199-7-3 PMC1413964

[20] Kiewe P, Gueller S, Komor M, Stroux A, Thiel E, et al (2009) Prediction of qualitative outcome of oligonucleotide microarray hybridization by measurement of RNA integrity using the 2100 Bioanalyzer capillary electrophoresis system. Ann Hematol 88: 1177–1183. 10.1007/s00277-009-0751-5 19424697

[21] Irizarry RA, Bolstad BM, Collin F, Cope LM, Hobbs B, et al (2003) Summaries of Affymetrix GeneChip probe level data. Nucleic Acids Res 31: e15 12582260 10.1093/nar/gng015 PMC150247

[22] Carvalho BS, Irizarry RA (2010) A framework for oligonucleotide microarray preprocessing. Bioinformatics 26: 2363–2367. 10.1093/bioinformatics/btq431 20688976 PMC2944196

[23] Kendziorski C, Irizarry RA, Chen KS, Haag JD, Gould MN (2005) On the utility of pooling biological samples in microarray experiments. Proc Natl Acad Sci U S A 102: 4252–4257. 15755808 10.1073/pnas.0500607102 PMC552978

[24] Kooperberg C, Aragaki A, Strand AD, Olson JM (2005) Significance testing for small microarray experiments. Stat Med 24: 2281–2298. 15889452 10.1002/sim.2109

[25] Smyth GK (2005) Limma: linear models for microarray data. In: Gentleman R, Carey V, Dudoit S, Irizarry R, Huber W, editors. Bioinformatics and Computational Biology Solutions using R and Bioconductor. Springer pp. 397–420.

[26] Huang dW, Sherman BT, Lempicki RA (2009) Systematic and integrative analysis of large gene lists using DAVID bioinformatics resources. Nat Protoc 4: 44–57. 10.1038/nprot.2008.211 19131956

[27] Fisher RA (1925) Statistical Methods for Research Workers. New York: Hafner

[28] Fisher RA (1948) Combining independent tests of significance. American Statistician 2: 30.

[29] Xiong DH, Liu XG, Guo YF, Tan LJ, Wang L, et al (2009) Genome-wide association and follow-up replication studies identified ADAMTS18 and TGFBR3 as bone mass candidate genes in different ethnic groups. Am J Hum Genet 84: 388–398. 10.1016/j.ajhg.2009.01.025 19249006 PMC2667986

[30] Rivadeneira F, Styrkarsdottir U, Estrada K, Halldorsson BV, Hsu YH, et al (2009) Twenty bone-mineral-density loci identified by large-scale meta-analysis of genome-wide association studies. Nat Genet 41: 1199–1206. 10.1038/ng.446 19801982 PMC2783489

[31] The WHI Study Group (1998) Design of the Women's Health Initiative clinical trial and observational study. The Women's Health Initiative Study Group. Control Clin Trials 19: 61–109. 9492970 10.1016/s0197-2456(97)00078-0

[32] Price AL, Patterson NJ, Plenge RM, Weinblatt ME, Shadick NA, et al (2006) Principal components analysis corrects for stratification in genome-wide association studies. Nat Genet 38: 904–909. 16862161 10.1038/ng1847

[33] Peng B, Yu RK, Dehoff KL, Amos CI (2007) Normalizing a large number of quantitative traits using empirical normal quantile transformation. BMC Proc 1 Suppl 1: S156 18466501 10.1186/1753-6561-1-s1-s156 PMC2367615

[34] Purcell S, Neale B, Todd-Brown K, Thomas L, Ferreira MA, et al (2007) PLINK: a tool set for whole-genome association and population-based linkage analyses. Am J Hum Genet 81: 559–575. 17701901 10.1086/519795 PMC1950838

[35] Li Y, Willer CJ, Ding J, Scheet P, Abecasis GR (2010) MaCH: using sequence and genotype data to estimate haplotypes and unobserved genotypes. Genet Epidemiol 34: 816–834. 10.1002/gepi.20533 21058334 PMC3175618

[36] Zhang L, Li J, Pei YF, Liu Y, Deng HW (2009) Tests of association for quantitative traits in nuclear families using principal components to correct for population stratification. Ann Hum Genet 73: 601–613. 10.1111/j.1469-1809.2009.00539.x 19702646 PMC2764806

[37] Devlin B, Roeder K (1999) Genomic control for association studies. Biometrics 55: 997–1004. 11315092 10.1111/j.0006-341x.1999.00997.x

[38] Willer CJ, Li Y, Abecasis GR (2010) METAL: fast and efficient meta-analysis of genomewide association scans. Bioinformatics 26: 2190–2191. 10.1093/bioinformatics/btq340 20616382 PMC2922887

[39] Higgins JP, Thompson SG, Deeks JJ, Altman DG (2003) Measuring inconsistency in meta-analyses. BMJ 327: 557–560. 12958120 10.1136/bmj.327.7414.557 PMC192859

[40] Schoppet M, Preissner KT, Hofbauer LC (2002) RANK ligand and osteoprotegerin: paracrine regulators of bone metabolism and vascular function. Arterioscler Thromb Vasc Biol 22: 549–553. 11950689 10.1161/01.atv.0000012303.37971.da

[41] Kalathur RK, Pinto JP, Hernandez-Prieto MA, Machado RS, Almeida D, et al (2014) UniHI 7: an enhanced database for retrieval and interactive analysis of human molecular interaction networks. Nucleic Acids Res 42: D408–D414. 10.1093/nar/gkt1100 24214987 PMC3965034

[42] Yang X, Khosravi-Far R, Chang HY, Baltimore D (1997) Daxx, a novel Fas-binding protein that activates JNK and apoptosis. Cell 89: 1067–1076. 9215629 10.1016/s0092-8674(00)80294-9 PMC2989411

[43] Wang Q, Xie S, Chen J, Fukasawa K, Naik U, et al (2002) Cell cycle arrest and apoptosis induced by human Polo-like kinase 3 is mediated through perturbation of microtubule integrity. Mol Cell Biol 22: 3450–3459. 11971976 10.1128/MCB.22.10.3450-3459.2002 PMC133784

[44] Liu H, Wang Y, Zhang Y, Song Q, Di C, et al (1999) TFAR19, a novel apoptosis-related gene cloned from human leukemia cell line TF-1, could enhance apoptosis of some tumor cells induced by growth factor withdrawal. Biochem Biophys Res Commun 254: 203–210. 9920759 10.1006/bbrc.1998.9893

[45] Chen Y, Sun R, Han W, Zhang Y, Song Q, et al (2001) Nuclear translocation of PDCD5 (TFAR19): an early signal for apoptosis? FEBS Lett 509: 191–196. 11741587 10.1016/s0014-5793(01)03062-9

[46] Shimizu S, Narita M, Tsujimoto Y (1999) Bcl-2 family proteins regulate the release of apoptogenic cytochrome c by the mitochondrial channel VDAC. Nature 399: 483–487. 10365962 10.1038/20959

[47] Shimizu S, Matsuoka Y, Shinohara Y, Yoneda Y, Tsujimoto Y (2001) Essential role of voltage-dependent anion channel in various forms of apoptosis in mammalian cells. J Cell Biol 152: 237–250. 11266442 10.1083/jcb.152.2.237 PMC2199613

[48] Kolbe D, Taylor J, Elnitski L, Eswara P, Li J et al (2004) Regulatory potential scores from genome-wide three-way alignments of human, mouse, and rat. Genome Res 14: 700–707. 15060013 10.1101/gr.1976004 PMC383316

[49] Wong BR, Besser D, Kim N, Arron JR, Vologodskaia M, et al (1999) TRANCE, a TNF family member, activates Akt/PKB through a signaling complex involving TRAF6 and c-Src. Mol Cell 4: 1041–1049. 10635328 10.1016/s1097-2765(00)80232-4

[50] Ramani AK, Bunescu RC, Mooney RJ, Marcotte EM (2005) Consolidating the set of known human protein-protein interactions in preparation for large-scale mapping of the human interactome. Genome Biol 6: R40 15892868 10.1186/gb-2005-6-5-r40 PMC1175952

[51] Perlman R, Schiemann WP, Brooks MW, Lodish HF, Weinberg RA (2001) TGF-beta-induced apoptosis is mediated by the adapter protein Daxx that facilitates JNK activation. Nat Cell Biol 3: 708–714. 11483955 10.1038/35087019

[52] Kawai T, Akira S, Reed JC (2003) ZIP kinase triggers apoptosis from nuclear PML oncogenic domains. Mol Cell Biol 23: 6174–6186. 12917339 10.1128/MCB.23.17.6174-6186.2003 PMC180930

[53] Golsteyn RM, Lane HA, Mundt KE, Arnaud L, Nigg EA (1996) The family of polo-like kinases. Prog Cell Cycle Res 2: 107–114. 9552388 10.1007/978-1-4615-5873-6_11

[54] Conn CW, Hennigan RF, Dai W, Sanchez Y, Stambrook PJ (2000) Incomplete cytokinesis and induction of apoptosis by overexpression of the mammalian polo-like kinase, Plk3. Cancer Res 60: 6826–6831. 11156373

[55] Jiang N, Wang X, Jhanwar-Uniyal M, Darzynkiewicz Z, Dai W (2006) Polo box domain of Plk3 functions as a centrosome localization signal, overexpression of which causes mitotic arrest, cytokinesis defects, and apoptosis. J Biol Chem 281: 10577–10582. 16478733 10.1074/jbc.M513156200

[56] Hughes DE, Wright KR, Uy HL, Sasaki A, Yoneda T, et al (1995) Bisphosphonates promote apoptosis in murine osteoclasts in vitro and in vivo. J Bone Miner Res 10: 1478–1487. 8686503 10.1002/jbmr.5650101008

[57] Weinstein RS, Chen JR, Powers CC, Stewart SA, Landes RD, et al (2002) Promotion of osteoclast survival and antagonism of bisphosphonate-induced osteoclast apoptosis by glucocorticoids. J Clin Invest 109: 1041–1048. 11956241 10.1172/JCI14538 PMC150947

[58] Weinstein RS, Jilka RL, Parfitt AM, Manolagas SC (1998) Inhibition of osteoblastogenesis and promotion of apoptosis of osteoblasts and osteocytes by glucocorticoids. Potential mechanisms of their deleterious effects on bone. J Clin Invest 102: 274–282. 9664068 10.1172/JCI2799 PMC508885

[59] Kameda T, Mano H, Yuasa T, Mori Y, Miyazawa K, et al (1997) Estrogen inhibits bone resorption by directly inducing apoptosis of the bone-resorbing osteoclasts. J Exp Med 186: 489–495. 9254647 10.1084/jem.186.4.489 PMC2199029

[60] Kousteni S, Bellido T, Plotkin LI, O'Brien CA, Bodenner DL, et al (2001) Nongenotropic, sex-nonspecific signaling through the estrogen or androgen receptors: dissociation from transcriptional activity. Cell 104: 719–730. 11257226

[61] Bush WS, Moore JH (2012) Chapter 11: Genome-wide association studies. PLoS Comput Biol 8: e1002822 10.1371/journal.pcbi.1002822 23300413 PMC3531285

[62] Richards JB, Zheng HF, Spector TD (2012) Genetics of osteoporosis from genome-wide association studies: advances and challenges. Nat Rev Genet 13: 576–588. 10.1038/nrg3228 22805710

[63] Liu YJ, Zhang L, Pei Y, Papasian CJ, Deng HW (2013) On genome-wide association studies and their meta-analyses: lessons learned from osteoporosis studies. J Clin Endocrinol Metab 98: E1278–E1282. 10.1210/jc.2013-1637 23783100 PMC3701269

[64] Benjamini Y, Hochberg Y (1995) Controlling the false discovery rate: a practical and powerful approach to multiple testing. J R Statist Soc B: 289–300.

