# Supplementary material for: Attenuated Monocyte Apoptosis, a New Mechanism for Osteoporosis Suggested by a Transcriptome-Wide Expression Study of Monocytes
Source: PLoS One. 2015 Feb 6;10(2):e0116792. doi: 10.1371/journal.pone.0116792 (PMC4319757; doi:10.1371/journal.pone.0116792)
Supplement: S1 Table — 1. Direction of regulation is the up- or down-regulation of a gene in the low BMD subjects. 2. Comparison group indicates the total, pre- or postmenopausal subgroups, where the differential expression is detected and replicated. 3. Meta-analysis p value was calculated by Fisher’s method [27,28] to combine the p values achieved in discovery cohort and replication cohort I. The genes in the table, 102 in total, are ranked by the meta-analysis p value. 4. PDCD5 is ranked at 7th. 5. VDAC1 is ranked at 17th (DOCX) [file pone.0116792.s001.docx]

| **Gene symbol** | **P value in discover cohort (n =73)** | **P value in replication cohort I (n =80)** | **Direction of regulation^1^** | **Comparison group^2^** | **Meta-analysis p value^3^** |
| --- | --- | --- | --- | --- | --- |
| SEMA6A | 7.61E-04 | 1.30E-04 | up | post | 1.70E-06 |
| CYP2W1 | 6.61E-05 | 2.55E-03 | down | total | 2.80E-06 |
| HDAC4 | 3.63E-05 | 9.82E-03 | down | total | 5.65E-06 |
| NCOA1 | 4.40E-03 | 8.22E-05 | up | pre | 5.73E-06 |
| CDH16 | 8.48E-04 | 5.41E-04 | up | total | 7.15E-06 |
| PHACTR1 | 1.81E-04 | 4.00E-03 | up | total | 1.10E-05 |
| ***PDCD5^4^*** | ***6.39E-05*** | ***1.42E-02*** | ***down*** | ***pre*** | ***1.35E-05*** |
| ACSL3 | 2.21E-04 | 4.19E-03 | up | pre | 1.38E-05 |
| DPP8 | 4.16E-03 | 2.60E-04 | up | pre | 1.59E-05 |
| PHACTR1 | 1.10E-03 | 1.00E-03 | up | post | 1.62E-05 |
| DDX18 | 3.66E-04 | 3.10E-03 | up | total | 1.67E-05 |
| SAPS3 | 8.26E-05 | 1.43E-02 | up | post | 1.73E-05 |
| DAXX | 1.44E-04 | 8.20E-03 | down | pre | 1.73E-05 |
| ANP32E | 4.38E-04 | 4.29E-03 | down | total | 2.67E-05 |
| KCNMB3 | 2.38E-04 | 8.29E-03 | up | post | 2.79E-05 |
| RAPGEF5 | 4.69E-04 | 4.49E-03 | up | post | 2.96E-05 |
| ***VDAC1^5^*** | ***8.32E-05*** | ***2.61E-02*** | ***down*** | ***total*** | ***3.04E-05*** |
| ABCG1 | 1.58E-04 | 1.62E-02 | up | total | 3.56E-05 |
| MAP4K1 | 5.84E-04 | 4.62E-03 | up | post | 3.73E-05 |
| SMARCA4 | 1.75E-03 | 1.55E-03 | down | pre | 3.76E-05 |
| COQ7 | 1.12E-03 | 2.62E-03 | up | total | 4.02E-05 |
| ZNF222 | 3.52E-04 | 8.76E-03 | up | post | 4.22E-05 |
| SPOCK3 | 6.03E-04 | 5.18E-03 | up | total | 4.27E-05 |
| TRIM3 | 4.12E-05 | 8.02E-02 | down | pre | 4.50E-05 |
| GRK6 | 4.73E-03 | 7.00E-04 | down | pre | 4.51E-05 |
| FAM155B | 1.01E-04 | 3.33E-02 | down | total | 4.58E-05 |
| GRIA1 | 3.79E-04 | 9.93E-03 | up | total | 5.08E-05 |
| SFRP1 | 4.82E-04 | 9.52E-03 | up | post | 6.10E-05 |
| KBTBD4 | 3.75E-03 | 1.25E-03 | up | pre | 6.23E-05 |
| NR2F2 | 9.45E-05 | 5.54E-02 | down | total | 6.89E-05 |
| USP25 | 3.37E-03 | 1.77E-03 | up | pre | 7.78E-05 |
| CREB5 | 2.03E-04 | 3.02E-02 | up | total | 7.97E-05 |
| CALCR | 8.28E-05 | 7.75E-02 | up | pre | 8.31E-05 |
| PIK3R4 | 4.30E-03 | 1.67E-03 | up | pre | 9.24E-05 |
| STXBP5L | 3.76E-04 | 1.91E-02 | up | total | 9.24E-05 |
| CDK10 | 5.18E-04 | 1.61E-02 | down | total | 1.06E-04 |
| EIF4A1 | 1.86E-04 | 4.55E-02 | down | pre | 1.07E-04 |
| DOCK4 | 1.06E-04 | 8.35E-02 | up | pre | 1.12E-04 |
| CDK10 | 9.21E-04 | 1.02E-02 | down | post | 1.18E-04 |
| NR2F2 | 1.08E-04 | 8.97E-02 | down | pre | 1.21E-04 |
| BCAM | 6.45E-04 | 1.68E-02 | up | total | 1.34E-04 |
| ALG13 | 2.76E-03 | 4.05E-03 | up | pre | 1.39E-04 |
| RABGGTB | 1.69E-04 | 6.68E-02 | up | post | 1.40E-04 |
| NFKB2 | 3.72E-03 | 3.24E-03 | down | pre | 1.49E-04 |
| TSPAN5 | 3.54E-04 | 3.41E-02 | up | post | 1.49E-04 |
| PAPSS1 | 3.39E-04 | 3.75E-02 | down | total | 1.56E-04 |
| ESR1 | 5.90E-04 | 2.18E-02 | down | post | 1.58E-04 |
| CASP1 | 2.79E-03 | 4.65E-03 | up | pre | 1.59E-04 |
| C21orf62 | 7.94E-04 | 1.72E-02 | up | post | 1.66E-04 |
| CLCN4 | 1.15E-03 | 1.32E-02 | up | post | 1.84E-04 |
| LYVE1 | 9.82E-04 | 1.65E-02 | up | post | 1.95E-04 |
| PKD1 | 2.17E-04 | 7.49E-02 | down | pre | 1.95E-04 |
| SNRPN | 3.72E-04 | 4.79E-02 | down | post | 2.13E-04 |
| MAPT | 1.15E-03 | 1.56E-02 | down | post | 2.14E-04 |
| LIMCH1 | 8.19E-04 | 2.29E-02 | down | total | 2.22E-04 |
| UBXN1 | 4.81E-03 | 4.95E-03 | down | pre | 2.77E-04 |
| FAM155B | 2.97E-04 | 8.77E-02 | down | post | 3.01E-04 |
| NFKBIA | 3.89E-03 | 7.09E-03 | down | pre | 3.17E-04 |
| FBXL2 | 7.71E-04 | 3.60E-02 | up | post | 3.19E-04 |
| GAS2L1 | 2.86E-03 | 1.04E-02 | down | pre | 3.41E-04 |
| TNFSF8 | 8.78E-04 | 3.48E-02 | up | pre | 3.49E-04 |
| FAM149B1 | 6.98E-04 | 4.74E-02 | up | total | 3.74E-04 |
| IER2 | 4.39E-03 | 7.65E-03 | down | pre | 3.80E-04 |
| TNIP1 | 8.08E-04 | 4.23E-02 | down | pre | 3.85E-04 |
| ANK2 | 7.80E-04 | 4.40E-02 | up | total | 3.87E-04 |
| C7orf23 | 3.70E-03 | 9.31E-03 | up | pre | 3.88E-04 |
| ATG3 | 1.12E-03 | 3.18E-02 | down | post | 4.01E-04 |
| HBP1 | 3.34E-03 | 1.28E-02 | up | pre | 4.73E-04 |
| CDH18 | 7.62E-04 | 6.05E-02 | up | post | 5.06E-04 |
| HUWE1 | 1.04E-03 | 5.46E-02 | up | post | 6.12E-04 |
| EPHB3 | 7.40E-04 | 8.31E-02 | down | total | 6.58E-04 |
| NEFL | 9.90E-04 | 6.84E-02 | up | post | 7.17E-04 |
| SNX6 | 2.87E-03 | 2.47E-02 | up | pre | 7.49E-04 |
| DYM | 4.96E-03 | 1.59E-02 | up | pre | 8.23E-04 |
| GOLGA2 | 2.20E-03 | 3.62E-02 | down | pre | 8.31E-04 |
| PPP1R15A | 3.39E-03 | 2.87E-02 | down | pre | 9.95E-04 |
| DENND3 | 2.31E-03 | 4.26E-02 | down | pre | 1.01E-03 |
| HMGA1 | 2.90E-03 | 3.65E-02 | down | pre | 1.08E-03 |
| MED14 | 4.69E-03 | 2.54E-02 | up | pre | 1.19E-03 |
| PPP1CC | 4.51E-03 | 2.84E-02 | up | pre | 1.28E-03 |
| HK3 | 2.81E-03 | 4.62E-02 | down | pre | 1.29E-03 |
| CUL1 | 2.29E-03 | 5.92E-02 | up | pre | 1.34E-03 |
| APOBEC3G | 4.90E-03 | 2.90E-02 | up | pre | 1.40E-03 |
| CDKN2D | 2.94E-03 | 5.11E-02 | down | pre | 1.47E-03 |
| LGALS8 | 3.54E-03 | 4.62E-02 | up | pre | 1.59E-03 |
| UBE4A | 4.42E-03 | 4.36E-02 | up | pre | 1.84E-03 |
| CYTH2 | 2.21E-03 | 9.22E-02 | down | pre | 1.93E-03 |
| FUT7 | 4.29E-03 | 5.50E-02 | down | pre | 2.21E-03 |
| PLK3 | 4.75E-03 | 5.55E-02 | down | pre | 2.43E-03 |
| CNOT7 | 2.98E-03 | 8.84E-02 | up | pre | 2.44E-03 |
| CNOT1 | 2.87E-03 | 9.42E-02 | up | pre | 2.49E-03 |
| NPAT | 4.45E-03 | 7.19E-02 | up | pre | 2.89E-03 |
| FUT4 | 4.21E-03 | 7.75E-02 | down | pre | 2.95E-03 |
| QRICH1 | 4.17E-03 | 7.90E-02 | up | pre | 2.97E-03 |
| STK38L | 4.82E-03 | 6.84E-02 | up | pre | 2.97E-03 |
| SH3BP2 | 3.91E-03 | 8.92E-02 | down | pre | 3.13E-03 |
| AK2 | 3.66E-03 | 9.63E-02 | up | pre | 3.16E-03 |
| AMZ2 | 4.40E-03 | 8.23E-02 | up | pre | 3.23E-03 |
| BMI1 | 4.36E-03 | 9.06E-02 | up | pre | 3.49E-03 |
| API5 | 4.79E-03 | 9.06E-02 | up | pre | 3.80E-03 |
| SLC26A6 | 4.61E-03 | 9.64E-02 | down | pre | 3.87E-03 |
| TRABD | 4.88E-03 | 9.85E-02 | down | pre | 4.15E-03 |
